# Migration of aluminum from food contact materials to food—a health risk for consumers? Part III of III: migration of aluminum to food from camping dishes and utensils made of aluminum

**DOI:** 10.1186/s12302-017-0117-x

**Published:** 2017-04-12

**Authors:** Thorsten Stahl, Sandy Falk, Alice Rohrbeck, Sebastian Georgii, Christin Herzog, Alexander Wiegand, Svenja Hotz, Bruce Boschek, Holger Zorn, Hubertus Brunn

**Affiliations:** 1 Hessian State Laboratory, Am Versuchsfeld 11, 34128 Kassel, Germany; 2 Hessian State Laboratory, Glarusstr. 6, 65203 Wiesbaden, Germany; 3 Institute of Food Chemistry and Food Biotechnology, Justus Liebig University Giessen, Heinrich-Buff-Ring 17, 35392 Giessen, Germany; 4 Institute of Medical Virology, Justus Liebig University, Schubertstraße 81, 35392 Giessen, Germany; 5 Hessian State Laboratory, Schubertstr. 60, 35392 Giessen, Germany

**Keywords:** Aluminum dishes, Aluminum camping utensils, Simulants, Foodstuffs, Release limits, Weekly intake

## Abstract

**Background:**

When cooking on a barbecue grill, consumers often use aluminum grill pans. For one, the pan catches the fats and oils that would drip into the embers causing the formation of potentially noxious smoke, and the pan also protects the food from being burned by direct heat from the coals. In addition, new aluminum products for use in ovens and grills are becoming increasingly popular. Due to their light weight and excellent heat transfer camping, utensils made of aluminum are, for example, often used by fishermen and mountain climbers. Preparing food in aluminum utensils can, however, result in migration of the aluminum to the foodstuffs.

**Results/Conclusions:**

In this study presented here, it was found that the transfer limit of 5.00 mg/L for aluminum is not exceeded using simulants for oil or for tap water; however, with an aqueous solution of 0.5% citric acid, the limit is clearly exceeded at 638 mg/L. This means that the Tolerable Weekly Intake (TWI) is exceeded by 298% for a child weighing 15 kg and for an adult weighing 70 kg it is equivalent to 63.8% of the TWI, assuming a daily uptake of 10 mL marinade containing lemon juice over a period of 1 week. Preparation of a fish dish with a marinade containing lemon juice in camping dishes would result in the TWI being exceeded by 871% for a child weighing 15 kg and by 187% for an adult weighing 70 kg assuming a daily uptake of 250 g over a period of 1 week.

## Background

A detailed summary of possible sources of exposure to aluminum, the release limits [2] for aluminum of 5.00 mg/kg or 5.00 mg/L foodstuff/beverage, the Tolerable Weekly Intake (TWI) of 1.00 mg aluminum per kg body weight and week [3], ranges of uptake values, as well as potential toxicological effects of aluminum can be found in part I [*exposure to aluminum, release of aluminum, Tolerable Weekly Intake* (*TWI*)*, toxicological effects of aluminum*] of this study. The present part (III) is devoted to the potential migration of aluminum to foodstuffs from dishes and camping utensils made of aluminum. Grill pans made of aluminum were tested using the food simulants tap water, olive oil, and 0.5% (w/v) aqueous solution of citric acid. Pureed ravioli and self-marinated fish patties were prepared in aluminum camping dishes. The marinade for the fish patties consisted of lemon juice and olive oil.

## Methods

A detailed description of sample preparation and analytical methods can be found in part I. Therefore, only details of experiments on the migration of aluminum from dishes and camping utensils to foods will be presented here.

### Aluminum dishes (cooking pans)

**Fig. 1:**
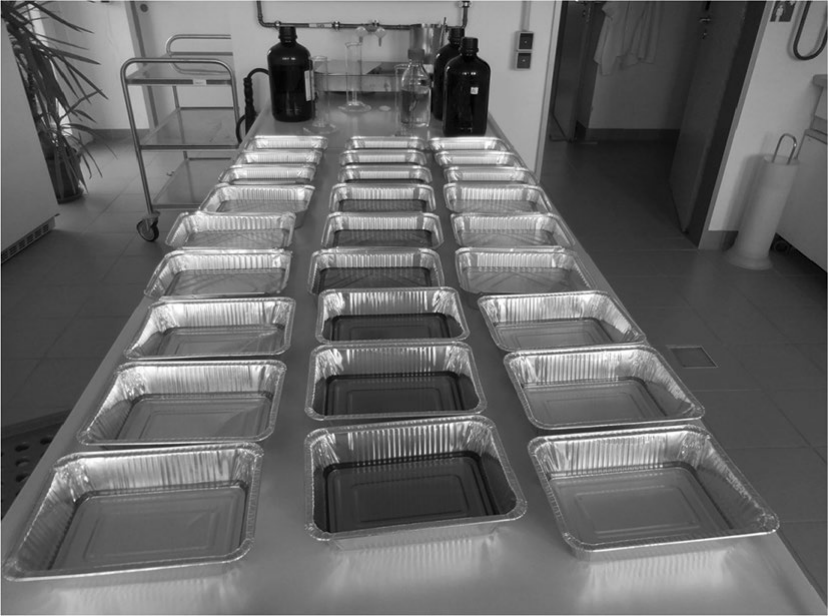
Aluminum pans filled with food simulants for testing [*left row* tap water, *middle row* olive oil, *right row* aqueous solution of 0.5% (w/v) citric acid]

In this series of tests, the migration of aluminum from cooking pans to three food simulants was analyzed (Fig 1). Three different brands of pans with capacities of 500 or 1000 mL were tested.

The simulants used for testing were water (pH = 7.58), water with 0.5% citric acid, and olive oil (pH = 5.80). The acid provides the conditions to determine the migration at a pH of <4.5 [4]. Water is the basis for studying migration in aqueous foodstuffs [2] with a pH >4.5 [4]. The third simulant used was olive oil. This was chosen to simulate food that naturally has a fat content and also to simulate potential marinades that contain oil, e.g., in the preparation of food cooked in the oven or on a grill. In order to reproduce typical consumer usage, the experiments were performed under three different conditions: short-term contact of 17 h overnight, long-term contact of 168 h, and heated to 160 °C for 2 h. It is assumed that the consumer generally fills the containers to only a fraction of the full capacity of 500 or 1000 mL so that a volume of 200 mL was chosen for the tests. After filling, the containers were covered with plastic-based microwave wrap (manufacturer: Melitta, Toppits^®^ brand, Germany) to avoid the unlikely, but potential contamination of the sample by the air in the laboratory and to minimize evaporation. Samples that were heated were additionally covered with a glass plate to reduce evaporation to a minimum. The conditions used in these experiments are summarized in Table 1.

**Table 1:** Description of the three different conditions used in testing aluminum pans

	Experimental conditions		
	Short-term contact	Long-term contact	Heated
Temperature	24 °C room temperature	24 °C room temperature	160 °C in a drying oven
Contact duration (h)	17	168	2
Simulated consumer activity	Preparation of a meal in aluminum dishes, e.g., marinating food overnight	Storage of food in aluminum dishes	Preparation of food in aluminum pans at high temperatures, e.g., in the oven or on a grill
Number of pans per test configuration	The number of aluminum pans used for each of the test conditions was as follows:		

At the end of the contact period (see Table 1)—or in the case of the heated samples, after cooling to room temperature—the simulants were transferred by glass funnel to a 250-mL sample bottle and subsequently tested for their aluminum concentration. Three samples of the simulants were transferred to a 250-mL sample bottle immediately following their preparation (lemon juice), after opening the bottle (olive oil), or after drawing (tap water) for blank value testing of aluminum. The individual blank values determined are listed in “Results”.

Blank value testing of the microwave wrap was performed by submersing the wrap in tap water in a 250-mL sample bottle overnight for 12 h to test for a potential migration of aluminum from the foil to water. After 12 h, the water was decanted and transferred to a 250-mL sample bottle for subsequent analysis.

### Camping utensils

**Fig. 2:**
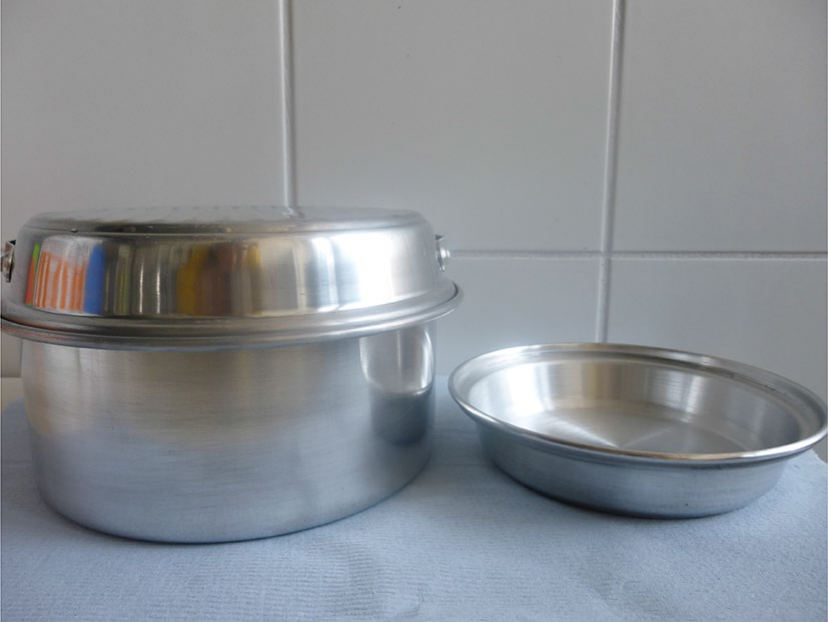
Pot with lid and pan made of aluminum were obtained from a supplier of trekking equipment

Reusable aluminum pots and pans were obtained from a supplier of trekking equipment (see Fig. 2) and were washed three times with tap water before testing.

### Tap water

The aluminum pots with a capacity of 1000 mL were filled with 500 mL tap water and covered with their appropriate lids and heated to 105 °C in a drying cabinet for 2 h. The pots were then allowed to cool to room temperature. The pans with a capacity of 300 mL were filled with 200 mL tap water and covered with microwave wrap and heated to 105 °C in a drying cabinet for 2 h. The pans were then allowed to cool to room temperature. Aliquots of 250 mL from each pot and pan were transferred to a 250-mL sample bottle for subsequent analysis.

### Olive oil^10^

The oil tested was labeled “Native olive oil Extra”. Three 20 mL samples of the oil were transferred to 30-mL polypropylene containers with screw lids (manufacturer: Genaxxon bioscience, Falcon^®^ brand). Pots and pans were filled with 100 mL oil and heated to 105 °C in a drying cabinet for 2 h. Pots were closed with their lids and the pans were covered with microwave wrap. The pots and pans were then allowed to cool to room temperature. The contents of the pots and pans were then transferred to 250-mL sample bottles for subsequent analysis.

### Canned ravioli (pasta pockets with a meat filling in tomato sauce^1^)

Two cans of ravioli were homogenized using an immersion blender with stainless steel blades. Three samples of this homogenate were frozen at −24 °C in 100-mL PE beakers with lids for blank value testing. Three aluminum pots were filled with the homogenized ravioli mass, covered with lids, and heated for 2 h at 105 °C in a drying cabinet. The pots were then allowed to cool to room temperature and the contents were transferred to 100-mL beakers with lids, and immediately frozen at −24 °C for subsequent analysis.

### Fish patties

**Fig. 3:**
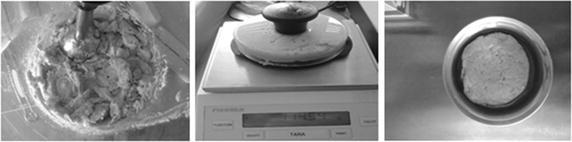
Preparation of the fish patties (*left* homogenizing the salmon filets, *middle* preparing the patties with a “burger press,” *right* fish patty in the aluminum pan before heating in the drying oven)

The contents of two packages of frozen salmon filets (250 g each) were homogenized using an immersion blender. Three samples of the homogenized fish were transferred to 100-mL PE beakers with lids and stored at −24 °C for subsequent blank value analysis. A marinade was prepared with olive oil and freshly pressed lemon juice: three pans (see Fig. 2) were filled with 20 mL olive oil and 8 mL lemon juice and shaken to mix the liquids. Three 20 mL blank value samples of the oil and lemon juice mixture were transferred to 100-mL PE sample bottles. The homogenized fish was formed into uniform patties (ca. 12 cm diameter and ca. 2 cm thick) using a plastic “burger press” (see Fig. 3, Weber, Ingelheim, Germany) and placed in the marinade in the pans. The three pans were covered with microwave wrap and heated for 2 h at 105 °C under standard conditions (Memmert, Schwabach, Germany). The pans were then allowed to cool to room temperature and the whole contents were frozen at −24 °C in 100-mL PE sample containers for subsequent analysis.

## Results

### Aluminum blank values of water and food

Samples that did not have contact with aluminum utensils (blank values) had low concentrations of aluminum: tap water 0.0007 mg/L, olive oil 0.201 mg/L, ravioli 2.47 mg/L or kg, in fish patties, including marinade 0.192 mg/kg and 0.5% (w/v) lemon juice below the limit of quantification (LOQ) of 0.025 mg/L. For the sake of clarity, all values shown below have been corrected for blank value before calculation of the aluminum concentration. The chemical blank values (*n* = 3) were found to be 0.005 ± 0.002 mg/kg and are therefore negligible in regard to aluminum concentration in the samples tested.

### Aluminum pans

A popular method of preparation for meat, fish, or vegetarian meals on the grill or in the oven is to place the food on aluminum pans or foil. This protects the food from direct heat radiation and prevents fat from dripping into the coals. In addition, many ready-to-eat meals are sold in aluminum dishes for direct preparation in the packaging. The question therefore arises whether aluminum migrates from the packaging or preparation dishes to the food.

#### Migration of aluminum in oil

**Fig. 4:**
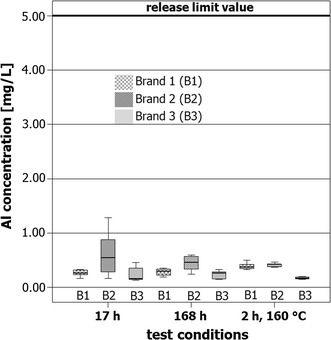
Box plots of the aluminum concentrations in oil samples after migration from the different brands 1, 2, und 3 of pans under the various experimental conditions 17 h contact (17 h) at room temperature, 168 h contact (168 h) at room temperature, and 2 h contact at 160 °C in the drying oven (2 h, 160 °C). The heavy line at 5.00 mg/L denotes the SRL [2]

Figure 4 shows the concentration of aluminum in oil after migration from the three different brands of aluminum pans after the various experimental conditions in the form of box plot diagrams.

As shown in Fig. 4, there are no obvious differences: the box plots of all three brands are comparable for all three experimental conditions. After heating for 2 h at 160 °C, however, a statistically significant difference is apparent. Brand 3 samples show a significantly lower (*p* < 0.05) aluminum concentration than the samples from brands 1 and 2. It must be noted, however, that all of the values measured are far below the Specific Release Limit (SRL) of 5.00 mg aluminum/L.

Thus, all brands complied with this limit under the experimental conditions chosen here, independent of the contact period or conditions in which the olive oil was in contact with the aluminum pans.

#### Migration of aluminum to water

**Fig. 5:**
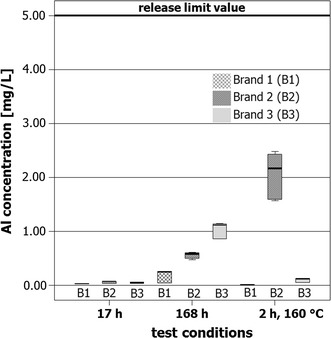
Box plots of the aluminum concentrations in water samples after contact with brands 1, 2, and 3 of aluminum pans after 17 h contact at room temperature (17 h), 168 h contact at room temperature (168 h) and 2 h contact at 160 °C in the drying oven (2 h, 160 °C). The heavy line at 5.00 mg/L denotes the SRL [2]

The aluminum concentration in the food simulant water varied from brand to brand of aluminum pan, from 0.009 mg/L (brand 1, experimental conditions 2 h contact at 160 °C) to 2.48 mg/L (brand 2, experimental conditions 2 h contact at 160 °C). The box plot diagram in Fig. 5 shows the aluminum concentrations for the three brands and the three different experimental conditions used in this test. The aluminum concentrations from the different grill pans with a contact period of 17 h were <0.2 mg/kg and for all brands in a comparable range. No significant differences (*p* > 0.05) were apparent between brands. As the contact period increased from 17 to 168 h at room temperature, there was an increase in aluminum concentration in tap water with all brands of pans. A significant (*p* < 0.05) difference between brands was also found in all pair-wise comparisons. With a contact period of 2 h at 160 °C, significant (*p* < 0.05) differences between brands were also seen in pair-wise comparisons. All concentrations measured were far below the SRL of 5.00 mg/L. Therefore, all brands of aluminum pans complied with this limit when in contact with water.

#### Migration of aluminum to citric acid

**Fig. 6:**
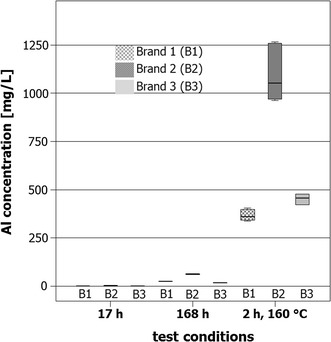
Box plots of the aluminum concentrations in citric acid samples after contact with brands 1, 2, and 3 of aluminum pans after 17 h contact at room temperature (17 h), 168 h contact at room temperature (168 h), and 2 h contact at 160 °C in the drying oven (2 h, 160 °C)

The box plot diagram in Fig. 6 shows the aluminum concentrations for citric acid from the three brands of aluminum pans and the three different experimental conditions used in this test. In contrast to Figs. 4 (test substance oil) and 5 (test substance water), the Y-axis was scaled to 1300 mg/L and consequently the heavy line marking the release limit at 5.00 mg/L was dispensed with. Aluminum concentrations varied between 0.149 mg/L (brand 3, contact period 17 h at room temperature) and 1266 mg/L (brand 2, contact period 2 h at 160 °C).

After a contact period of 17 h, the aluminum concentration in citric acid from the individual pans was comparable; however, all pair-wise comparisons showed statistically significant differences (*p* < 0.05). After a contact period of 168 h, differences were also significant between brands in pair-wise comparisons (*p* < 0.05). Significant (*p* < 0.05) brand-dependent differences were greatest after a 2-h contact period at 160 °C: aluminum concentrations were the highest in samples from brand 2 pans. In regard to the SRL, it should be noted that all three brands complied with this limit for a contact period of 168 h (values between 16.9 and 61.7 mg/L). The SRL was clearly exceeded by all three brands of pans when the citric acid was heated to 160 °C for 2 h (values between 405 and 1266 mg/L). Since aluminum concentrations for oil and water were comparable, the arithmetic mean was calculated for all results per simulant oil and water (brand independent) as shown in Table 2. Because of the large differences with citric acid as food simulant, the brands of pans are presented individually (brand-dependent), as well as the arithmetic mean of all results (brand independent) are shown in Table 2. Based on these data, the aluminum uptake and percentage to TWI reached were calculated for a child weighing 15 kg and an adult weighing 70 kg (Table 2). A volume of 500 mL water was assumed as the daily intake. For the simulants oil and 0.5% citric acid, it was assumed that 10 ml of each in a marinade would be consumed with the food processed in the aluminum pans.

**Table 2:** Aluminum uptake for a child/adult and the percentage to TWI

Foodstuff	Experimental conditions	Mean concentration (mg/L)^a^	Aluminum uptake for a child (mg/week)^b^	Percentage to TWI child	Aluminum uptake for an adult (mg/week)^c^	Percentage to TWI adult
Olive oil	17 h (*n* = 9)	0.007	0.026	0.172	0.026	0.037
	168 h (*n* = 9)	0.006	0.022	0.148	0.022	0.032
	2 h, 160 °C (*n* = 9)	0.006	0.023	0.150	0.023	0.032
Water	17 h (*n* = 9)	0.042	0.147	0.980	0.147	0.210
	168 h (n = 9)	0.589	2.07	13.7	2.07	2.95
	2 h, 160 °C (n = 9)	0.725	2.54	16.9	2.54	3.63
Citric acid pan brand 1	17 h (*n* = 3)	0.368	0.026	0.172	0.026	0.037
	168 h (*n* = 3)	25.2	1.76	11.8	1.76	2.52
	2 h, 160 °C (*n* = 3)	367	25.7	171	25.7	36.7
Citric acid pan brand 2	17 h (*n* = 3)	3.12	0.218	1.456	0.218	0.312
	168 h (*n* = 3)	61	4.27	28.5	4.27	6.1
	2 h, 160 °C (*n* = 3)	1094	76.6	511	76.6	109
Citric acid pan brand 3	17 h (*n* = 3)	0.165	0.012	0.077	0.012	0.017
	168 h (*n* = 3)	16.9	1.18	7.9	1.18	1.69
	2 h, 160 °C (*n* = 3)	452	31.6	211	31.6	45.2
Citric acid (mean)	17 h (*n* = 9)	1.21	0.085	0.564	0.085	0.121
	168 h (*n* = 9)	34.3	2.4	16	2.4	3.42
	2 h, 160 °C (*n* = 9)	638	0.045	298	44.7	63.8

^a^Arithmetic mean of the results for triplicate tests of all three brands of aluminum pans

^b^The results shown are for a child weighing 15 kg based on a daily portion of water (500 mL) or a 10 mL portion of olive oil or citric acid over a period of 1 week (7 days)

^c^The results shown are for an adult weighing 70 kg based on a daily portion of water (500 mL) or a 10 mL portion of olive oil or citric acid over a period of 1 week (7 days)

Consuming 10 mL olive oil daily^2^ after a contact period of 17 h would result in reaching a maximum of 0.172% TWI for a child weighing 15 kg and a maximum of 0.037% for an adult weighing 70 kg. Consuming 500 mL water daily would result in reaching a maximum of 16.9% TWI (2 h, 160 °C) for a child weighing 15 kg and a maximum of 3.63% (2 h, 160 °C) for an adult weighing 70 kg. Consuming 10 mL 0.5% citric acid daily^3^ would result in reaching a mean value of 298% TWI (2 h, 160 °C) for a child weighing 15 kg and 63.8% (2 h, 160 °C) for an adult weighing 70 kg.

### Camping utensils

**Fig. 7:**
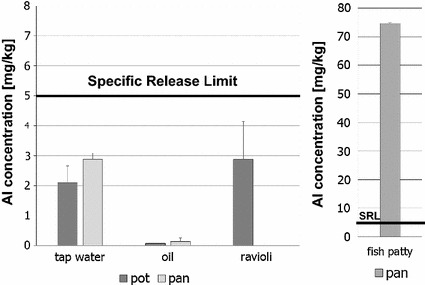
Aluminum concentration in foodstuffs (water, oil, ravioli, and fish patties) after a contact period of 2 h at 105 °C in pots and pans made of aluminum. The heavy line at 5.00 mg/L denotes the SRL [2]

All results presented here are the arithmetic mean of the concentrations determined in triplicate experiments. Olive oil, after 2 h at 105 °C in a pot, was found to have an aluminum concentration of 0.08 mg/L, whereas oil that had been in a pan for 2 h at 105 °C had an aluminum concentration of 0.139 mg/L. After 2 h contact in a pot tap water had an aluminum concentration of 2.11 mg/L and in a pan the concentration was 2.88 mg/L. After preparation of the ravioli in a pan, the aluminum concentration was 2.88 mg/L. The highest concentration of aluminum was detected in the fish patties at 76.6 mg/L (Fig. 7).

It must be noted that in these experiments with a maximal value of 2.88 mg/kg (tap water in pan or ravioli), the SLR is complied with. In the case of the fish patties, however, the SRL is exceeded by a factor of 15. Based on the arithmetic mean of all results for oil, water, ravioli and fish patty, the percentage to TWI has been calculated for a child weighing 15 kg and an adult weighing 70 kg (Table 3).

**Table 3:** Aluminum uptake for a child/adult and percentage to TWI (values rounded)

Foodstuff	Experimental conditions	Mean concentration (mg/L)	Aluminum uptake child^a^ (mg/week)	Percentage to TWI child	Aluminum uptake adult^b^ (mg/week)	Percentage to TWI adults
Oil	Pots (*n* = 3)	0.078	0.005	0.036	0.005	0.008
	Pans (*n* = 3)	0.136	0.01	0.063	0.01	0.014
Water	Pots (*n* = 3)	2.11	7.39	49.2	7.39	10.6
	Pans (*n* = 3)	2.88	10.1	67.2	10.1	14.4
Ravioli	Pots (*n* = 3)	2.88	5.05	33.6	5.05	7.2
Fish patty	Pans (*n* = 3)	74.6	131	871	131	187

^a^Results are for a child weighing 15 kg based on a daily uptake of 10 mL oil or of 500 mL water or 250 g ravioli or fish patties for a period of 1 week (7 days)

^b^Results are for an adult weighing 70 kg based on a daily uptake of 10 mL oil or 500 mL water or 250 g ravioli or fish patties for a period of 1 week (7 days)

Daily consumption of 10 mL olive oil^4^ under the conditions listed here would result in reaching a maximum of 0.063% (pan) to TWI for a child weighing 15 kg and 0.014% (pan) to TWI for an adult. A daily uptake of 500 mL water would result in reaching a maximum of 67.2% for a child weighing 15 kg and a maximum of 14.4% for a 70 kg adult. A daily uptake of 250 g ravioli would result in reaching a maximum of 33.6% for a child weighing 15 kg and a maximum of 7.2% for a 70 kg adult. Daily consumption of 500 g of fish patties would exceed the TWI by 871% for a 15 kg child and by 187% for a 70 kg adult.

## Discussion

In health evaluation no. 033/2007 [1], the German Federal Institute for Risk Assessment (BfR) clearly states that there is “No danger of contracting Alzheimer’s disease from aluminum in household utensils.” Furthermore, this document states that there is no scientific evidence indicating a connection between aluminum uptake from foodstuffs, including drinking water, pharmaceuticals, or cosmetics and Alzheimer’s disease. No increases in the frequency of amyloid plaques in the brain have been found in dialysis patients or in aluminum workers, both groups of people with extensive contact with aluminum. The BfR, therefore, does not recognize a health danger for consumers through aluminum uptake from food and cooking utensils or cosmetics [1]. The BfR does recommend that consumers avoid the use of aluminum pots or dishes for acidic or salted foodstuffs such as apple sauce, rhubarb, tomato puree, or salt herring due to the increased solubility of aluminum under the influence of acids and salts, thus prophylactically avoiding the “unnecessary ingestion” of aluminum [1].

In this present study, aluminum household utensils such as (grill) pans and camping utensils were tested in regard to release of aluminum to foodstuffs. In some instances, extreme “worst-case conditions” were intentionally chosen, such as the use of water with 0.5% citric acid and aluminum grill pans or acidic marinades in camping utensils made of aluminum.

To summarize it can be said that:

The use of aluminum grill pans may result in an additional aluminum exposure that is not negligible for the consumer if acidic marinades are used. The specific release limit (SRL) was not exceeded with any of the grill pans using water and oil. The SRL was exceeded with all grill pans if they are subjected to 0.5% citric acid for 17 h with values ranging from 16.9 to 61.7 mg/L. If the temperature is increased to 160 °C for a contact period of 2 h, the concentrations measured were even higher (from 405 to 1266 mg/L).

- The use of camping utensils may result in an additional aluminum exposure that is non-negligible. Although the SRL is not exceeded by the use of water and oil or the preparation of ravioli for a 15 kg child, 33.6% of the TWI may be reached. Daily consumption of 250 g fish patties prepared with a lemon juice marinade may result in an SRL of 74.6 mg/L and thus clearly exceed the TWI by 187% for an adult or 871% for a child.
- The daily uptake of ravioli and fish patties represents a worst-case scenario. If these dishes are consumed once per week at the given concentrations, a child weighing 15 kg and consuming the ravioli will reach 4.8% TDI. An adult consuming 250 g fish patties once per week will reach 26.7% and a child 124% TDI.

## Footnotes

1 List of ingredients—tomato sauce: water, tomato paste (26.4%), modified starch, iodized salt, sugar smoked bacon fat (bacon, smoke), sunflower oil, spices, wine vinegar with estragon extract (wine vinegar, aroma), flavor enhancer monosodium glutamate, celery, herbs. List of ingredients—ravioli: durum wheat semolina, breading mixture (wheat flour, yeast, iodized salt), pork, wheat gluten, sunflower oil, bacon, wheat flour, semolina, spices, iodized salt, yeast extract, vegetable oil egg white powder, egg yolk powder, herbs, lactose, flavoring, sugar.

2 It can be considered highly unlikely that a person would consume 10 mL olive oil daily over a period of 1 week so that this calculation represents a worst-case situation.

3 It can be considered highly unlikely that a person would consume 10 mL 0.5% citric acid daily over a period of 1 week so that this calculation represents a worst-case situation.

4 It must be considered highly unlikely that a person would consume 10 mL olive oil daily over a period of 1 week, but this is assumed here for the sake of comparison to water.
